# It's not just in my head: An intersectional, social and systems‐based framework in gender and sexuality diversity

**DOI:** 10.1111/papt.12438

**Published:** 2022-11-09

**Authors:** Brendan J. Dunlop, James Lea

**Affiliations:** ^1^ Division of Psychology and Mental Health The University of Manchester Manchester UK

**Keywords:** gender diversity, intersectionality, LGBT, LGBTQ, mental health, sexuality diversity

## Abstract

**Background:**

The mental health and well‐being of gender and sexuality diverse (GSD) people needs to be understood within a socio‐political and cultural context.

**Aims:**

In this paper, an intersectional, social and system‐based framework for understanding the mental health and well‐being of GSD people is presented, for practitioners within this field to consider GSD mental health experiences and challenges within context.

**Materials and Methods:**

Starting with a consideration of the current landscape of understanding, pivotal theories and understandings within the field are outlined. The need for a framework that centralises intersectionality and broader systemic considerations is presented.

**Results:**

The framework provided has an explicit focus on four key features: (1) intersectionality, (2) institutions, policies and laws, (3) people and groups and (4) social stories.

**Discussion:**

Consideration of each of these ‘circles of influence’ can help practitioners to understand the multi‐layered and intersectional experience of GSD folk and allows for an understanding of potential intervention at both an individual and systemic and societal level.

**Conclusion:**

Use of such a framework in practice goes above and beyond what is currently available by centralising the role and impact of such wider systemic variables through an intersectional lens. The framework can be applicable worldwide given its flexibility to consider and apply pertinent policies, laws, people, groups and social stories within a particular country or culture.

**Practitioner points**

- The mental health of gender and sexuality diverse people needs to be considered within a broader socio‐political context.
- Practitioners can utilise the intersectional, social and systems‐based framework presented in the paper to inform clinical work.
- Understanding broader systemic variables, and the impact these have on individual mental health, provides additional avenues for intervention.
- Future research into gender and sexuality diversity should consider systemic variables within research design, data collection and analysis to holistically capture pertinent information.

## INTRODUCTION

The mental health and well‐being of people who identify as Gender and Sexuality Diverse (GSD) needs to be understood within social, political and cultural context (Dunlop, 2022a). Such challenges to wellbeing are often connected to systems, structures and stories much bigger than individual people. Within this article, the term GSD will be primarily used as an umbrella term to capture individuals who identify as belonging to the Lesbian Gay, Bisexual, Trans, Queer, Asexual, Intersex and/or Transgender and Gender Non‐Conforming (TGNC) minoritised communities.

In this narrative conceptual article, the authors will outline a framework that they believe will be useful when thinking clinically about mental health for GSD populations. This paper will begin by providing some important definitions, before moving on to outline the current landscape of understanding for GSD mental health and wellbeing. After this, the importance of an intersectional (Crenshaw, 1989, 1991) lens will be discussed. What follows is the authors' perspective on an Intersectional, Social and Systems‐based (InSoS) Framework of GSD mental health and wellbeing. Some of the initial ideas for this model were germinated in *The Queer Mental Health Workbook* (Dunlop, 2022a). The framework proposed is based on the understanding that humans do not exist in isolation, and the ‘trickle down’ effect of systemic variables (such as policies and laws) can affect the way in which people and groups think, feel and behave. For GSD people this can lead to external and internal stressors that can create psychological distress that interact with mental health and wellbeing. This article will conclude with a clinical vignette illustrating how the InSoS framework would be useful in supporting practitioners and researchers to think about and understand the difficulties of people who identify as GSD.

This article will go above and beyond what is currently available within the literature by systematically recognising the compounding impact of wider systems, structures and stories on individual mental health and wellbeing. Whilst other prominent theories in this arena (such as Minority Stress Theory; Meyer, 1995) describe the impact of stressors on individual mental health and wellbeing and understand the importance of external variables, they can decentralise the important role of wider societal, cultural and political variables. Furthermore, whilst Intersectionality theory (Crenshaw, 1989, 1991) is considered an important facet of minoritised experience, to the authors' knowledge, no particular theory or framework marries together intersectional considerations with wider systemic variables, to describe the unique mental health challenges this can lead to for GSD people. Given that GSD people all over the world are living under greater or lesser degrees of political, cultural and societal expectation/pressure, the framework presented here can account for differences from country to country by spotlighting particular laws, policies or practices within that cultural context that can get into our heads and insidiously ‘trickle down’ to directly impact upon individual wellbeing and experiences of distress.

Sexuality diversity refers to the broad range of sexualities that exist. Whilst a plethora of nuance exists within human sexuality, the following sexuality definitions may be useful to consider. They include *homosexuality* (an antiquated term) *and/or gay/lesbian* (attraction to the same gender), *bisexuality* (attraction to multiple genders), *heterosexuality and/or Straight* (attraction to the assumed ‘opposite’ sex and gender), *asexuality* (absence of sexual attraction to any gender) and *pansexuality* (attraction to people regardless of their gender identity and/or sexuality).

Gender diversity is an umbrella term used to incorporate TGNC people, which is a term used in academic literature to refer to trans and non‐binary individuals (American Psychological Association, 2015). The term transgender is used for people whose sex assigned (or assumed) at birth and gender identity do not correspond in the expected manner. The term non‐binary is as an umbrella term to refer to people whose gender identity or expression does not fit within the gender binary of male/man or female/woman, and as such they may prefer to use gender neutral pronouns, for example they/them (Gosling et al., 2022a; Richards et al., 2016; Thorne et al., 2019). In contrast, the term cisgender means having a gender identity that normatively relates to sex assigned at birth, in a socially constructed context where the normative assumption is that penis and testes equates to male, which equates to boy/man, and vulva and ovaries equates to female and thus equates to girl/woman (Gosling et al., 2022a).

The identifier ‘queer’ is an antiquated term that has often been weaponised against GSD folk and used as a derogatory slur, given that this term is synonymous with ‘strange’. Increasingly, people within GSD communities are beginning to reclaim this term and use it as a self‐identifying category: typically referring to a GSD identity outside of the societal norm and potentially representing a multitude of gender and/or sexuality and/or relationship expression. Use of this term is unique and individual, and the authors recognise that some people will be averse to such a term given the historical, interpersonal and societal trauma attached to this label.

### Current landscape of understanding

It is by now well‐established that GSD people experience disproportionate mental health difficulties (Prell & Traeen, 2018; Ross et al., 2018) compared with heterosexual (‘straight’) people (Semlyen et al., 2016). Increased psychological distress, low mood (Reisner et al., 2016) self‐harm (Gosling et al., 2022a) and suicidality (Gosling et al., 2022b) exists compared with cisgendered peers. People who identify as GSD also often encounter structural institutional heterosexism and homo/bi/transphobia within health services (Bartlett et al., 2001; Lea et al., 2010; McFarlane, 1998).

A prominent theory related to GSD peoples' experience of oppression is the Minority Stress Theory (Meyer, 1995, 2003, 2010, 2015). Meyer et al. (2021) suggest that negative mental health outcomes could be explained in the context of Minority Stress. GSD minoritised groups navigate their minoritised identity within a heteronormative and cisnormative world (Valentine & Shipherd, 2018). Minority Stress Theory suggests that GSD people are confronted with distal (external) and proximal (internal) processes and minority stressors that confer risk for mental health difficulties. Such stressors could include victimisation, institutionalised or individual discrimination based on sexuality or gender (e.g. hate crimes and lack of inclusive policies, such as the recent government position on conversion therapy in the UK: Government Equalities Office, 2021), internalised homo/bi/transphobia, rejection expectation, stigma and identity concealment. Thus, trying to live in a world that suggests you are wrong, or bad or should not exist, understandably creates psychological distress and mental health challenges (Gosling et al., 2022a; Testa et al., 2015). A provocative and necessary illustration of Minority Stress Theory, and the impact it has, comes from Dominic Davies (1998):

My mother tongue is “Gay”, and I think, feel, and behave more spontaneously and naturally in that language. When I am in the “country” of heterosexuals, then everything I think, say, and do has to go through an interior translator; this can reduce my spontaneity especially with emotions, and result in my being quite guarded and defensive.

(Davies, p. 117)

Whilst growing up GSD in a heteronormative and cisnormative world creates inherent challenges and pain, it may also create opportunities, strength and resilience in people. Within Minority Stress Theory, the impact of these minority stressors is believed to be reduced by individual and community factors, which can act as a buffer against psychological distress. For example, personal qualities and characteristics, community resilience, finding one's ‘tribe’, affirming and accepting groups, belonging and social support (Gosling et al., 2022b; Meyer, 2003, 2015; Sattler et al., 2016; Testa et al., 2015).

Lesbian, gay and bisexual individuals thrive when they have supportive social networks, accept their emotions and process them with insight, and view the future with hope and optimism…(allowing) individuals to thrive despite societal prejudice.

(Kwon, 2013, p. 379)

Whilst positive, it unfortunately situates the solution and inoculation of a sociocultural problem on the resources of the individual and those belonging to GSD communities, rather than society at large. This is risky for people who may not always have the resources, due to other psychological, social or emotional factors. It is important to remember how difficult it can be for someone to live and openly embrace their GSD identity. This path requires vulnerability and strength in the world, which is often a difficult tension to balance.

Meyer's theory was extended and expanded upon by Hatzenbuehler (2009). Hatzenbuehler proposed the Psychological Mediation Framework to understand how ‘sexual minority stigma gets under the skin’ (Hatzenbuehler, 2009, p. 707). He proposed that the relationship between minority stress and mental health challenges is a result of people being confronted with minority‐related stigma in the social world, that then leads to minority stress, which then in turn leads to elevations in emotional dysregulation, difficulties with social and interpersonal relationships and certain cognitive processes, such as rumination (Hatzenbuehler et al., 2009a, 2009b; Lewis et al., 2014). It is the effect of these ‘mediating’ psychological and emotional variables that then confer risk for mental health difficulties, rather than a direct causative effect of the minority stress. This mediational effect is also recognised within the Pachankis et al. (2020) intra‐minority gay community stress model, a model specifically focussed upon gay and bisexual men. By focussing upon socially based stressors within the gay and bisexual community (that have links to wider narratives of masculinity and position within society) such as status, competition and sexual gain, mental health difficulties can be predicted.

Another important consideration is that of the ‘weathering hypothesis’. Described by Geronimus (1992) originally in relation to variation in birth outcomes between those of different ethnicities, this hypothesis suggests that cumulative socioeconomic disadvantage has an effect on the health of Black African American women. When considered for GSD populations, the same hypothesis is plausible: cumulative minority stress could be a factor in the mental and physical health outcomes of GSD groups (Hood et al., 2019). Evidently, focussing upon external stressors, variables and influences is pertinent to understanding the mental health experiences and subsequent distress of GSD people.

## PRESENTING AN INTERSECTIONAL, SOCIAL AND SYSTEMS‐BASED FRAMEWORK IN GSD

Current theoretical understandings including Minority Stress Theory (Meyer, 1995, 2003, 2015, Meyer et al., 2021; Testa et al., 2015), the Psychological Mediation Framework (Hatzenbuehler, 2009), Intra‐minority gay community stress (Pachankis et al., 2020) and the weathering hypothesis (Geronimus, 1992) all have a common denominator: they all recognise external, social and relational influences as having a significant bearing on individual distress and mental health. It could be argued that ‘from a radical queer perspective people inhabit and experience the disturbance and distress that has been given to them by the world around them’ (Lea, 2020, p. 16).

Wilson and Cariola's (2020) systematic review of qualitative studies identified policy and environment as one of the core themes of GSD youth mental health and it has furthermore been recognised that GSD‐specific affirmative therapies need to incorporate wider environmental and systemic perspectives (see Carvalho et al., 2022; Cohen & Feinstein, 2020) so as to not dismiss or invalidate real‐world threats to safety and wellbeing. This dismissal and invalidation can often present itself subtly in the consulting room. For example, a practitioner labelling a GSD persons' experience as hypervigilance and paranoia, when in fact the behaviour itself is functional and appropriate vigilance to maintain safety in the world. The addition of the prefix ‘hyper’ invalidates and others a GSD person's experience, and disregards real social and systemic threats. The authors argue that to decentralise a social, systemic and systems‐based understanding of GSD mental health is at best risking invalidation of experience and at worst actively harming by neglecting contextual, institutional and systemic trauma.

*The Queer Mental Health Workbook* (Dunlop, 2022a), upon which this article is broadly based, presents the ‘circles of influence’ as a way of understanding this myriad of social, systemic, historical and contemporary external factors that contribute to psychological distress and poor mental health for GSD people. Drawing strongly upon influences such as Bronfenbrenner's Ecological Systems Theory (Bronfenbrenner, 1977), and similar to the conceptual model of contextual influences by Russell and Fish (2016), attention is drawn to human existence as being an experience that necessitates relation to other(s).

This article aims to address the above considerations explicitly, by developing the InSoS Framework. The authors draw the readers' attention to thinking about intersectionality and three broad social and systemic ‘circles of influence’ (Dunlop, 2022a) around them: (1) institutions, policies and laws, (2) people and groups, and (3) social stories, all of which can enact and confer distal and proximal stressors, ‘trickling down’ to have an impact upon GSD peoples' mental health and wellbeing. The intersectional considerations (Dunlop & Lea, 2022) and circles of influence from Dunlop (2022a) provide the backdrop and golden thread for the framework presented in this paper.

### Intersectional, social and systems‐based framework in GSD: intersectionality

The authors present intersectionality as the linchpin to the InSoS framework in GSD. The term intersectionality was developed by Professor Kimberlé Crenshaw (1989, 1991), an Black American feminist, civil rights advocate and leading scholar. She created the term ‘intersectionality’ to describe the complex experience of occupying the junction of multiple marginalised identities, which creates a more nuanced and complicated experience of oppression and discrimination.

Audre Lorde is relevant to this discussion of intersectionality, as she utilised her life and creativity to confront a range of socially created and delivered injustices of racism, heterosexism, sexism, classism and homophobia:

there is no such thing as a single issue struggle, because we do not lead single issue lives.

(Lorde, 1984, p. 147).

GSD people living with multiple marginalised intersecting identities are not living single Cook et al. (2019) issue lives. As practitioners and researchers there is an imperative to understand and create ideas and theories to house their experience, based in science. This is because intersectionality links to Minority Stress Theory, the Psychological Mediation Framework and the weathering hypothesis, as all identities are connected to systems of privilege and oppression that give or take away peoples' power. For example, navigating the social landscape as a gay man may be difficult. When you are a Black neurodivergent trans woman, there are multiple ways in which your experiences of living in a White, heteronormative, cisnormative, patriarchal and ableist world can independently and as a complex collective, create unique experiences of psychological stress and distress (Dunlop & Lea, in press). Dunlop and Lea (2022) provide a set of activities focused upon understanding intersectionality that researchers and practitioners can utilise in their own practice with clients.

Holding in mind the important notion of intersectionality, power and privilege, the authors will discuss the three broad circles of influence through a GSD lens. There will be necessary cross‐over between considerations described below in the three circles of influence. Whilst presented here separately, each circle can be thought of as existing within the next; influencing and influenced by each other.

### Intersectional, social and systems‐based framework in GSD: institutions, policies and laws

Whether we are consciously aware of it or not, our existence is shaped, governed and influenced by culture and specific laws and policies that are enacted within the territory and cultural history we live. It is recognised that laws, policies, practices and social stories will be explicitly different country to country, and so the impact these have to a greater or less extent will vary for GSD people. Such top‐down laws and policies are usually widespread and applicable to whole societies or sections of societies, though can have disproportionate impacts upon certain groups. This article will highlight some considerations particularly from a United Kingdom (UK) perspective.

It has been well established for decades now that a strong relationship exists, for example, between socioeconomic deprivation and mental health outcomes (Drukker & van Os, 2003; Fernández‐Niño et al., 2014; Gunnell et al., 1995). In the UK, austerity policies (specific governmental decisions to reduce public expenditure) brought in by the Conservatives and Liberal Democrats coalition government from 2010 onwards reduced funding to some mental health services and reduced financial welfare benefits. For those that had mental health difficulties and needed support with this, the availability of service provision reduced. Others saw their benefits payments reduced, which can also significantly impact on psychological wellbeing (Mattheys, 2015). A broad systemic influence—namely policies and laws—can have a very real effect on individual mental health.

Policies and laws have directly impacted people who express sexuality diversity for centuries. In 1533 the Buggery Act outlawed anal sex between men and was punishable by death (British Library, 2022). In 1861, the Act was downgraded, and within Section 61 of the Offences Against the Person Act (1861) anal sex was punishable instead by life imprisonment.

Often the historical and institutional abuses targeted against GSD people are invisible and pernicious. One such example relates to the Nazi state and Holocaust, where more than 100,000 men identified as homosexual were arrested by the state, with around 15,000 of these people sent to concentration camps (United States Holocaust Memorial Museum, 2021). Even when the camps closed, many gay men remained in prison as homosexuality was illegal in Germany. It is pertinent to note that many other political prisoners of the camps could apply for support, whilst homosexual men could not make themselves visible. Men returning home were shunned by families due to shame and stigma, and their stories were not valued by Holocaust research (Jensen, 2021). It is worth noting that a clear misogynistic stance has made the history of Lesbians in the Holocaust even less visible, though there is new research describing similar experiences of these women (Hájková, 2020). Akin to the weathering hypothesis, the insidious effects of historical oppression and violence is in the social fabric that envelops GSD people, creating a backdrop from which present‐day struggles can be traced back to.

Up until the implementation of the Sexual Offences Act (1967), homosexuality remained criminalised in England and Wales. For Scotland, the Criminal Justice Act (1980) saw homosexuality decriminalised, and The Homosexual Offences Order (1982) saw the same for Northern Ireland. In 2022, there are still 71 countries worldwide that criminalise homosexuality (Human Dignity Trust, 2022). If an individual's identity is the point of state‐sanctioned punishment, then it is going to perhaps be unsurprising if that person feels anxious, threatened, low in mood or even suicidal. Indeed, in a longitudinal epidemiological survey study, Hatzenbuehler et al. (2010) observed significant increases in psychiatric diagnosis in adults residing in states that enacted same‐sex marriage bans. For example, diagnosis of Generalised Anxiety Disorder rose 248.2% in lesbian, gay and bisexual people living in such states. Whilst associative in nature, data like this does suggest that policies and laws can impact directly and/or indirectly upon individual mental health; a distal stressor as described by Minority Stress Theory.

Because of such laws and policies, men historically underwent a variety of physical and psychological so called ‘conversion therapies’ to alter their sexuality. Psychological so‐called ‘conversion therapies’ still exist in the UK, and at the time of writing, proposed laws to ban this do not include so called ‘conversion therapies’ for TGNC people. A recent evidence synthesis has concluded that there is no robust evidence that ‘conversion therapy’ can change an individual's gender identity or sexuality and is instead often associated with harm (Government Equalities Office, 2021).

Other UK policies such as Section 28 of the Local Government Act (1988) stated that local authorities (and by virtue, teachers, schools and other educational institutions) should not ‘intentionally promote homosexuality or publish material with the intention of promoting homosexuality’ or ‘promote the teaching in any maintained school of the acceptability of homosexuality as a pretended family relationship’. In the years following the implementation of Section 28, young LGBTQIA+ people received the explicit message that the core of who they were was not to be taught or even spoken about. During formative years, this sense of shame and internalised minority stress contributes towards difficulties with self‐esteem, self‐compassion and self‐acceptance (Dunlop, 2022a; Gilbert & Irons, 2009). It was only in 2003 when Section 28 was fully repealed (Stonewall, 2022). Many people in their 20s, 30s and up would likely not recall being taught about diversity of sexuality or gender identity throughout their educational career.

Institutions, such as educational establishments, health care services, social services or religious organisations, can often directly and indirectly affect the mental health of minoritised people. Perhaps unsurprisingly, structural racism within institutions has a significant impact upon the mental health of Black, Indigenous and People of Colour (Kalin, 2021). Indeed, the recent and ongoing COVID‐19 pandemic highlighted structural homo‐, bi‐ and transphobia within the US health care system, given that public health surveillance is often disorganised, haphazard or lacking for minoritised groups such as sexuality and gender minority people (Sell & Krims, 2021). Disease profiling and subsequent health care interventions are therefore focused upon communities for which data exists. This same principle applies to the mental health of LGBTQIA+ people. Because homophobia is often embedded within cultural systems, institutions, religion and politics, effective intervention should be targeted at such levels (Ventriglio et al., 2021).

An important reason for understanding the historical and cultural landscape that has shaped the GSD community of today, is that these historical and generational traumas (Kelly et al., 2020) are passed down in the minds, hearts and psychologies of contemporary GSD people from those that came before. The narratives from historical and generational traumas reify what is allowed and permissible in society:

- They regulate the rules by which GSD people relate with others
- They regulate the emotions that can be shared
- They regulate GSD peoples' attention and the need to be vigilant for safety
- They ultimately intensify the experiences of shame and internalised oppression for GSD people, leading to self‐policing and censorship that actually does the oppressor's work
- They demand the need for concealment and assimilation of GSD people into the heterosexist and cisgendered world
- They dictate how GSD people need to stay safe, for example the need to ‘pass’ as straight or cisgendered
- They shape how GSD people experience and internalise the heterosexism and cissexism in society.

It is also important in this area to hold in mind the importance of race, age, ethnicity, disability, culture and religion as intersecting identities for GSD people, which creates more complex forms of oppression and more nuanced psychologies to understand. For example, it would be imperative to understand the social, cultural and religious context of GSD people migrating to the UK from countries with particularly oppressive state‐sanctioned discrimination of GSD people, for example Russia, a selection of African countries, countries in the Middle East etc. These people may even fear discussing their GSD identity with health professionals, which is an important consideration to be held in mind when working clinically with such folk. Their ‘reluctance’ to engage may represent a firmly held safety strategy, especially if health professionals in their home country would report aspects of their identity to government officials.

The British Psychological Society (2019) guidelines for working with GSD people stipulate that practitioners should maintain awareness of the impact that the socio‐political climate can have on mental health and wellbeing. Some practitioners may wonder how they can assimilate such institutional or cultural considerations within their work with GSD people. Questions that can be used to inform this particular circle of influence could include the following, adapted from Dunlop (2022a):

- If you are religious, does your religion or faith accept your GSD identity? If not, are there other denominations within your religion or faith that do accept this?
- If you are part of a sports club, do your teammates accept you? If not, what do you do to cover up or hide the parts of yourself you do not want them to see? At what cost do you do this?
- Are there any clubs, groups or organisations that you want to join, but have not yet done so? Is this connected to your GSD identity?
- Are there any clubs, groups or organisations that you are a part of that celebrate being GSD? Are you able to fully embrace your identity when in these spaces?
- Are there laws or policies that exist to keep your identity hidden? What do you do to cover up or hide the parts of yourself that could jeopardise your safety? At what cost do you do this?

### Intersectional, social and systems‐based framework in GSD: people and groups

As humans rarely live in total isolation from others, it is important to consider proximal and distal people and groups in an individual's life, to ascertain the impact of such people on cumulative minority stress. Especially pertinent for children and young people will be the caregivers, adults, siblings and peers around them, and by extension, those peoples' thoughts, feelings, attitudes and behaviours towards GSD experience and identity. Frommer (1995) describes the experience of an outsider, whereby ‘…the (gay) child is most often an alien within his family… (and) often adopts the identity of an outsider even before he can label the nature of his difference’ (p. 78). This sense of not belonging can create a strong feeling of being excluded and othered in groups (Dalal, 2006). The heterosexism, cisnormativity and homo/bi/transphobia that is exerted within society (and baked into our social fabric, as described in relation to institutions, policies and laws above) becomes crystallised internally, leading to shame and distress (Lea, 2020).

Consider two examples of an 18‐year‐old person who thinks they may identify as non‐binary. The first person's family, peers and social groups embraces their gender identity as being non‐binary. The caregiver helps to facilitate their access to support groups and other young people that share this experience. This caregiver takes the time to research gender non‐conformity online and communicates to their child that they may make mistakes when using pronouns, though they are learning and trying their best.

Let us consider a second example. This is one of an unsupportive, anxious and possibly hurtful caregiver, family, peers and social groups. Gender diversity and identifying as non‐binary is not something that this caregiver has any experience of and is scared to learn about. They were brought up to think that people were men or women, and that there is no other option. The persons' access to the internet is restricted in the home in case going online ‘puts ideas in their head’.

Based on the above information, it could be speculated that despite broader challenges connected to gender diversity, the young adult of the first caregiver is likely to have these socially created challenges buffered or ‘moderated’ by their supportive caregiver(s). Their caregiver has made it clear that they are a safe base from which they can explore this part of their identity, and they are a safe pair of hands to receive them if things get tricky for them. Contrast this with the second caregiver, and one can imagine a child that may experience compounded mental health challenges given the unsupportive home environment in which they are living. As described in the Psychological Mediation Framework, such home‐based stressors may confer risk for other general psychological processes such as emotional dysregulation or cognitive rumination, mediating the effect and impact of minority stress. Any stressors this person may face outside the home life will not be mitigated against by the protective factor of caregiver support that the first person has, and we can hypothesise that they are likely to experience multiple, and perhaps more complex experiences of psychological distress as a result. If this person was to present to mental health services with low self‐esteem, self‐harmful behaviours and emotional dysregulation, a focus upon their intrapersonal processes divorced from their home context is not going to address the core difficulty they are experiencing. Thought challenging or self‐esteem building here may be akin to a chocolate umbrella in the sun: temporary precarious protection or relief that quickly melts away when ‘outside’ in the real world.

Evidently, the people and groups that GSD people are (or are not) connected to are important considerations within the formulation of their difficulties. Some questions that may ascertain the link between GSD mental health and people and groups could include:

- *Who is important in your life? Why/why not?*
- *Who are you closest to?*
- *Who do you go to for support when you need help?*
- *What do the important people in your life think of your sexuality/gender identity?*
- *Who negatively impacts your wellbeing (and in what ways)?*
- *In an ideal world, who would you like to distance yourself from?*
- *Which sport/religious/cultural/community group(s) do you belong to?*
- *How do these groups appraise your identity?*
- *Do you feel safe in these group(s)? In what ways? Why/why not?*
- *Do you feel you need to hide aspects of yourself? If so, what, why and how?*
- *Do you feel worried that people may treat you differently? If so, why and how?*

The following questions adapted from Dunlop (2022a) and Gosling et al. (2022a) may also be useful to consider:

- *Do you know many GSD people? If so, what do you like about them and what do you dislike about them? Is that the type of person you want to be?*
- *If you do not know many GSD people, would you like to?*
- *Is there someone famous who is also GSD that you admire? Why?*
- *Is being GSD accepted or celebrated by the people or groups you hang around with?*
- *Is there anybody that you know that really brings out and celebrates the GSD parts of your identity?*
- *Is there anybody that you really want to invite into your world, but you worry that they will reject you? Why do you think they will reject you?*
- *Does you sex assigned (assumed) at birth match your gender identity? How does this feel in your family, social group, school or work?*
- *How would you identify your gender? How does this feel in your family, social group, school or work?*
- *What pronouns would you like to use?*
- *Do people mis‐gender you? How does that feel?*
- *Do you feel that you belong? If so, where and why? If not, where and why?*

An exploration of an GSD person's identity and experience without reference to the important people and groups in their lives is likely to miss important facets of their relational experience. Focusing a therapy or therapeutic intervention therefore at a purely individual level is likely to (at best) invalidate their experience and (at worst) potentially miss an abusive caregiver or possible safeguarding concern. Conversely, discussion of an GSD person's identity in relation to others can be a powerful way of delineating aspects of themselves they want to embrace more (or less) and how this experience interacts with their overall wellbeing.

### Intersectional, social and systems‐based framework in GSD: social stories

From the institutions, policies and law that govern us, to the people and groups that experience such implicit and explicit rules, social stories develop. Drawing upon concepts from Narrative Therapy (White & Epston, 1990), social stories can be powerful influences on individual mental health and wellbeing. Social stories (or social narratives) can be thought of as information that is assumed to be true about certain people or groups, based on and influenced by the prevailing societal norms of the time. A prominent and recent example of this are the social stories associated with the HIV and AIDS crisis of the 1980s. Human Immunodeficiency Virus—HIV—was first described as a ‘rare cancer seen in 41 homosexuals’ (New York Times, 1981) that can develop into Acquired Immune Deficiency Syndrome (AIDS). This particular article (one of the first to report on the HIV crisis) goes on to state that:

According to Dr. Friedman‐Kien, the reporting doctors said that most cases had involved homosexual men who have had multiple and frequent sexual encounters with different partners, as many as 10 sexual encounters each night up to four times a week… Many patients also reported that they had used drugs such as amyl nitrite and LSD [lysergic acid diethylamide, colloquially known as “acid”] to heighten sexual pleasure. (p. 20).

Social narratives soon followed that the promiscuity of gay men was linked to this disease, and as HIV at the time mostly affected gay and bisexual men, the media labelled this as ‘the gay plague’ (Ruel & Campbell, 2006). This prevailing narrative led to significant shame, marginalisation and rejection by loved ones. The stigma that is still associated with HIV and AIDS today can be traced back and linked to prominent narratives that were borne from the public panic of the 1980s and 1990s. We are beginning to see the same seeds of social shame blooming once again with the current Monkeypox outbreak, which is affecting predominantly gay and bisexual men.

Social stories are really important when considering minoritised mental health. Important questions adapted from Dunlop (2022a) that may allow GSD people to think about this circle of influence may include:

- What social stories exist about GSD people in your community? Are they positive or negative?
- Have these social stories had any impact on the way you have embraced, or rejected, your GSD identity? Why?
- If you could create a social story from scratch about being GSD, what would it be and why?
- If we lived in a world free from discrimination, what parts of your identity would you celebrate? Are there any of these parts that you feel able to connect with in the society that you currently live in?

## INTERSECTIONAL, SOCIAL, AND SYSTEMS‐BASED FRAMEWORK IN GSD: CLINICAL CASE EXAMPLE

Let us use an example, adapted from Dunlop (2022b). Krystyna is 42 years old and originally from Poland. Krystyna is a lesbian woman and lost two fingers on her right hand in a workplace accident in Poland many years ago. She came to the UK 11 years ago, with her partner Zofia. Krystyna is currently experiencing low mood, feelings of anxiety and difficulties in her relationship with Zofia.

### Institutions and laws

Institutional influences (such as the Catholic Church) may have fed into law or policy changes, and indeed education and curricula within Poland. The absence of visibility when growing up in school and any laws or cultural differences connected to homosexuality in Poland may be important considerations. Indeed, a central reason for leaving Poland may have been because of Krystyna and Zofia's fears for their safety, given their non‐heterosexual relationship.

### People and groups

These factors can influence the people and groups around Krystyna too. Perhaps work colleagues or friends hold certain prejudiced ideas about some aspects of her identity, such as her sexuality. Perhaps they enact such prejudicial behaviours by excluding her from social events or making comments about her ‘lifestyle choice’. Perhaps Krystyna has internalised some of this prejudice (otherwise known as ‘internalised homophobia’) and has come to dislike these parts of herself; maybe even projecting some of this dislike onto Zofia. Whether Krystyna's colleagues have been talking about her or not, Krystyna may have learned to become very vigilant to any perceived threat in order to keep herself and others safe, because of persecution in their home country.

### Social stories

If we consider the wider system and environment that Krystyna has been living in, we may identify narratives connected to multiple minoritised aspects of her identity. For example, social stories exist around women (and their position in the world compared to men), sexuality (homosexuality being considered ‘lesser than’, or deviant from, heterosexuality, with particular pertinence perhaps to the legacy of the Nazi state on cultural norms in this part of Europe) disability (in particular the value of aesthetics) and migration (a non‐native of a country being considered ‘lesser than’ a native).

### Intersectionality

Any one of the above circles of influence could impact upon mental health and well‐being. When we consider the multiple marginalised identities (Crenshaw, 1989) that Krystyna holds, we can recognise these are creating unique and complex experiences of oppression. For example, the adverse experiences she has within her workplace in the UK means that Krystyna must manage expectations and prejudices that people hold about women, being lesbian, being disabled and also being a migrant, all at once. Krystyna may have to (un)consciously manage this compounded oppression; she has more reasons to be vigilant and wary when around others compared to a heterosexual, able‐bodied, UK‐born man. Even if Krystyna was able to foster a more positive or resilient understanding of her homosexuality, she would still have to navigate experiences of oppression at a social and systemic level for other aspects of her identity, which is tiring and exhausting. Thus, considering Krystyna's presenting difficulties of low mood and anxiety, and her relationship difficulties with Zofia, these feelings seem to make much more sense once we have considered the context that Krystyna is living in, and the intersectional experience of oppression she has had to navigate and will have to continue navigating.

## CONCLUSION

The authors believe that the Intersectional, Social and Systems‐based (InSoS) framework in GSD provides a unique psychological model and therapy approach that explicitly focuses on intersectionality, social and systemic factors as integral to clinical assessment, formulation and an integral agent of change for GSD people experiencing distress. The InSoS framework provides practitioners with a framework to think about and act in prevention and intervention at multiple levels, including individual, systemic and societal (Dunlop et al., 2020, 2021), by helping both client and practitioner to understand broader and more distal factors involved in the development and maintenance of difficulties. By using such a framework and considering their difficulties through this particular lens, clients may feel less self‐blame and a deeper awareness of the role of context in the formation and maintenance of their difficulties. This framework goes above and beyond what is already available by centralising the role and impact of such wider systemic variables through an intersectional lens and can be applicable to GSD people worldwide given the flexibility of application, dependent on the pertinent laws, policies, people, groups, social stories, etc. within a particular country or culture. The InSoS framework could equally help researchers to consider social and systemic factors that may be important to focus upon, control for or include within data collection and/or analysis.

The authors believe that the InSoS framework can act as an alternative conceptualisation of distress in GSD people from the usual western, patriarchal and neoliberal context that demands difficulties belong to the individual and lives inside their heads. The authors believe that the InSoS framework conceptualises psychological distress in people who identify as GSD as developing and being maintained in relationships, families, communities and societies that are heteronormative and cisnormative. It is the assertion that this model can help practitioners and people who identify as GSD to understand difficulties relationally, systemically and socially to recognise and reflect that the challenges and experiences of GSD people are ‘not just all in their heads’, and are part of a much more complex historical, systemic and relational landscape.

## AUTHOR CONTRIBUTIONS

**Brendan J. Dunlop:** Conceptualization; writing – original draft; writing – review and editing. **James Lea:** Writing – original draft; writing – review and editing.

## CONFLICT OF INTEREST

All authors declare no conflict of interest.

## Data Availability

No data has been included in this article and therefore no data is available.
